# Deletion of Gtpbp3 in zebrafish revealed the hypertrophic cardiomyopathy manifested by aberrant mitochondrial tRNA metabolism

**DOI:** 10.1093/nar/gkz218

**Published:** 2019-03-27

**Authors:** Danni Chen, Zengming Zhang, Chao Chen, Shihao Yao, Qingxian Yang, Feng Li, Xiao He, Cheng Ai, Meng Wang, Min-Xin Guan

**Affiliations:** 1 Division of Medical Genetics and Genomics, The Children's Hospital, Zhejiang University School of Medicine, Hangzhou, Zhejiang 310058, China; 2 Institute of Genetics, Zhejiang University School of Medicine, Hangzhou, Zhejiang 310058, China; 3 Department of Human Genetics, Zhejiang University School of Medicine, Hangzhou, Zhejiang 310058, China; 4 Key Laboratory of Reproductive Genetics, Ministry of Education, Zhejiang University, Hangzhou, Zhejiang 310058, China; 5 Joint Institute of Genetics and Genome Medicine between Zhejiang University and University of Toronto, Hangzhou, Zhejiang 310058, China

## Abstract

GTPBP3 is a highly conserved tRNA modifying enzyme for the biosynthesis of τm^5^U at the wobble position of mitochondrial tRNA^Glu^, tRNA^Gln^, tRNA^Lys^, tRNA^Trp^ and tRNA^Leu(UUR)^. The previous investigations showed that *GTPBP3* mutations were associated with hypertrophic cardiomyopathy (HCM). However, the pathophysiology of GTPBP3 deficiency remains elusively. Using the *gtpbp3* knockout zebrafish generated by CRISPR/Cas9 system, we demonstrated the aberrant mitochondrial tRNA metabolism in *gtpbp3* knock-out zebrafish. The deletion of *gtpbp3* may alter functional folding of tRNA, indicated by conformation changes and sensitivity to S1-mediated digestion of tRNA^Glu^, tRNA^Lys^, tRNA^Trp^ and tRNA^Leu(UUR)^. Strikingly, *gtpbp3* knock-out zebrafish displayed the global increases in the aminoacylated efficiencies of mitochondrial tRNAs. The aberrant mitochondrial tRNA metabolisms impaired mitochondrial translation, produced proteostasis stress and altered activities of respiratory chain complexes. These mitochondria dysfunctions caused the alterations in the embryonic heart development and reduced fractional shortening of ventricles in mutant zebrafish. Notably, the *gtpbp3* knock-out zebrafish exhibited hypertrophy of cardiomyocytes and myocardial fiber disarray in ventricles. These cardiac defects in the *gtpbp3* knock-out zebrafish recapitulated the clinical phenotypes in HCM patients carrying the *GTPBP3* mutation(s). Our findings highlight the fundamental role of defective nucleotide modifications of tRNAs in mitochondrial biogenesis and their pathological consequences in hypertrophic cardiomyopathy.

## INTRODUCTION

Transfer RNA (tRNA) molecules from all living organisms contain modified nucleotides, which are derivatives of four nucleotides adenosine (A), guanosine (G), uridine (U) and cytidine (C). To date, more than 110 species of modifications have been identified in tRNAs from various organisms (1,2). These modified nucleotides play critical roles in the structure and function of tRNAs, including the stabilization of anticodon structure, codon recognition at the decoding site of small rRNA and the improvement of reading frame maintenance (3–8). Of these, the nucleotide at position 34 (wobble position of anticodon) of tRNA is more prone to be modified than those at other positions of tRNAs (5,9,10). In *Escherichia coli*, the uridine at position 34 of tRNA is always modified, specifically, the nucleoside 5-methyl-aminomethy-2-thio-uridine (mnm^5^s^2^U34) occurs at the wobble position 34 of bacterial tRNA^Glu^, tRNA^Lys^ and tRNA^Gln^ (11,12). The nucleotides at position 34 of mammalian mitochondrial tRNAs carry the diverse species of modifications including 5-formylcytidine (f^5^C), 5-taurinomethyluridine (τm^5^U) and 5-taurinomethyl-2-thiouridine (τm^5^s^2^U) (4,13–16). The biosynthesis of τm^5^s^2^U modification was catalyzed by highly conserved tRNA modifying enzymes GTPBP3, MTO1 and TRMU (14,17–19). In human mitochondrion, MTO1 and GTPBP3 are involved in the formation of the 5-taurinomethyl group of tRNA^Glu^, tRNA^Gln^, tRNA^Lys^, tRNA^Trp^ and tRNA^Leu(UUR)^, while TRMU catalyzes the thiolation at position 2 of U34 in tRNA^Glu^, tRNA^Gln^, tRNA^Lys^ (17,19–23).

The deficient modifications at U34 of mitochondrial tRNAs have been linked to human diseases (10,23–25). The defective τm^5^U of tRNA^Leu(UUR)^ was responsible for mitochondrial encephalopathy lactic acidosis and stroke-like episodes (MELAS), while the loss of τm^5^s^2^U of tRNA^Lys^ was associated with myoclonic epilepsy associated with ragged red fibers (MERRF) (26–27). The deficient τm^5^s^2^U modification caused by mutations in the *TRMU* and *MTO1* genes were responsible for mitochondrial dysfunctions leading to clinical phenotypes, including deafness, reversible infantile liver failure, hypertrophic cardiomyopathy, and lactic acidosis (28–31). In particular, GTPBP3 deficiency was associated with hypertrophic cardiomyopathy, lactic acidosis, and encephalopathy (32–34). The inactivation of GTPBP3 in human cell lines led to the deficient taurine modification at U34 in mitochondrial tRNA^Glu^, tRNA^Gln^, tRNA^Lys^, tRNA^Trp^ and tRNA^Leu(UUR)^, reduced efficiency of mitochondrial translation and the deficiency of OXPHOS (14,32,35). Furthermore, the defective τm^5^U34 modification and mitochondrial translations were observed in mutant cell lines carrying the *GTPBP3* mutations, derived from patients of families with hypertrophic cardiomyopathy (HCM) characterized by myocardial and myocyte hypertrophy in left ventricle (14,32). However, the lack of animal disease model makes the pathophysiology of GTPBP3 deficiency elusively. In our previous investigation, *gtpbp3* knockdown zebrafish embryos displayed the marked decrease of mitochondrial ATP generation and defective development (36). To further investigate the pathophysiology of GTPBP3 deficiency, we generated a *gtpbp3*^ko^ zebrafish model produced by genome editing using the CRISPR/Cas9 system. First, the *gtpbp3*^ko^ zebrafish was assessed for the *in vivo* effects of defective *gtpbp3* on mitochondrial tRNA metabolism, mitochondrial translation, proteostasis stress and enzymatic activities of electron transport chain complexes. The *gtpbp3*^ko^ zebrafish was further evaluated for the effect on cardiac function including cardiac looping and ventricular fractional shortening, the size of cardiomyocyte, the expression of the cardiac natriuretic peptides as well as the ultrastructure of cardiac myofibrils.

## MATERIALS AND METHODS

### Experimental fish and maintenance

AB wild-type strain and myocardium-specific transgenic *Tg* (*cmlc2*: *egfp*) zebrafish (*Danio rerio*) were used for this investigation. The animal protocols used in this investigation were approved by the Zhejiang University Institutional Animal Care and Use Committee. All fish were kept in recirculating water at 28°C and fed with commercial pellets at a daily ration of 0.7% of their body weight. Embryos were reared at 28.5°C according to standard protocols (37). Embryos were staged by hours post fertilization (hpf) and days post fertilization (dpf) (38).

### Gtpbp3-Knockout zebrafish line generation by CRISPR/Cas9 system

The zCas9 expression plasmid pSP6–2sNLS-spCas9 was linearized by *Xba I* and used as a template for Cas9 mRNA *in vitro* synthesis with mMESSAGE mMACHINE mRNA transcription synthesis kits (Ambion). The sequence of sgRNAs was designed according to criteria as described previously (39). The gRNA transcription plasmid was pT7-gRNA. We used the CRISPR/Cas9 design tool (http://zifit.partners.org) to select specific targets to minimize off-target effects. Cas9-encoding mRNA (300 ng/μl) and gRNA (200 ng/μl) were co-injected into one-cell-stage wild-type embryos. Injected embryos were incubated at 28.5°C, and collected for making genomic DNA for genotyping at 50 hpf. Genomic DNA of the 50 hpf injected embryo was used as template to amplify *gtpbp3* gene by using the following two primers: F1: GTTGTTGTTTGAGGAGAA; R1: TAAAAGACTGGAGCATTG. The fragments were cloned by using the TA Cloning Kit (TAKARA) and then were sequenced.

### Mitochondrial tRNA analysis

Total RNAs were isolated from fish using Totally RNA™ Kit (Ambion, Inc). The presence of thiouridine modification in the tRNAs was verified by the retardation of electrophoretic mobility in a polyacrylamide gel that contains 0.05 mg/ml (*N*-acryloylaminophenyl) mercuric chloride (APM) (19,29,40,41). Total RNAs were separated by polyacrylamide gel electrophoresis and blotted onto positively charged membrane (Roche Applied Science). Each tRNA was detected with the specific digoxigenin (DIG)-oligodeoxynucleoside probe at the 3′ termini as detailed elsewhere (40,41). Oligodeoxynucleosides used for DIG-labeled probes were zebrafish mitochondrial tRNA^Glu^, tRNA^Gln^, tRNA^Lys^, tRNA^Trp^, tRNA^Leu(UUR)^, tRNA^Ala^ and 5S rRNA as detailed elsewhere (Supplemental Table S1) (42,43). The oligodeoxynucleotides were generated by using DIG-oligonucleotide Tailing kit (Roche). APM gel electrophoresis, the hybridization and the quantification of 2-thiouridine modification in tRNAs were conducted as detailed (19,29,40,41).

For tRNA Northern blot analysis, 5 μg of total RNAs were electrophoresed through a 10% polyacrylamide gel without (native gel) or with (denature gel) 8 M urea in Tris–borate–EDTA buffer (TBE) (after heating the sample at 65°C for 10 min), and then electroblotted onto a positively charged nylon membrane for the hybridization analysis with DIG-labeled oligodeoxynucleotide probes. Oligodeoxynucleoside probes for mitochondrial tRNA^Glu^, tRNA^Gln^, tRNA^Lys^, tRNA^Trp^, tRNA^Leu(UUR)^, tRNA^Ala^ and 5S rRNA were described as above. The hybridization and quantification of density in each band were performed as detailed previously (7,44–46).

For tRNA aminoacylation analysis, 5 μg of total RNA were electrophoresed at 4°C through an acid (pH 5.2) 10% polyacrylamide/8 M urea gel to separate the charged and uncharged tRNA as detailed elsewhere (46,47). The gels were electroblotted onto a positively charged nylon membrane (Roche) for the hybridization analysis with oligodeoxynucleotide probes specific tRNA^Lys^, tRNA^Trp^, tRNA^Leu(UUR)^, tRNA^Tyr^, tRNA^Met^ and tRNA^Ala^, as detailed elsewhere (42). Quantification of density in each band was performed as detailed previously (46,47).

The S1 nuclease cleavage analysis was performed as detailed elsewhere (48). In brief, 2 μg of total RNAs were used for the cleavage reaction in the presence of 1 μg/μl total yeast tRNA and 1U/μl S1 nuclease (Thermofisher) in the 5 μl reaction buffer containing 40 mM sodium acetate (pH 4.5), 300 mM NaCl and 2 mM ZnSO4. Reaction mixtures were incubated at 28°C for indicated times and quenched by adding 5 μl loading buffer. Samples were electrophoresed through a 10% denaturing polyacrylamide gel with 8 M urea and then electroblotted onto a positively charged nylon membrane for hybridization analysis with 3′end DIG-labeled oligodeoxynucleotide probes as described above.

### Western blotting analysis

Western blotting analysis was performed as detailed elsewhere (42,44,49). Fish were sacrificed after anesthesia and homogenized in RIPA reagent (Invitrogen) using a homogenizer. Twenty micrograms of total cellular proteins were electrophoresed through 10% bis-Tris SDS-polyacrylamide gels and then transferred to a polyvinyl difluoride (PVDF) membrane. The antibodies used include anti-Gtpbp3 (Sigma, HPA042158), anti-Nd5 (Abcam, ab92624), anti-Nd6 (Abcam, ab81212), anti-Cytb (ABclonal, A9762), anti-Co2 (Proteintech, 55070-1-AP), anti-Sdha (Proteintech, 14865-1-AP), anti-Atp5c (Proteintech 60284-1-Ig), anti-Tfam (Proteintech, 19998-1-AP), and anti-Tufm (Proteintech, 26730-1-AP), anti-Yars2 (Abcam, ab228957), anti-Kars (Proteintech, 14951-1-AP), anti-Afg3l2 (Proteintech, 14631-1-AP), anti-Clpp (Proteintech, 15698-1-AP) and anti-Gapdh (Sigma, SAB2701826). Peroxidase AffiniPure goat anti-mouse IgG and goat anti-rabbit IgG (Jackson) were used as secondary antibodies and protein signals were detected using the ECL system (CWBIO). Quantification of density in each band was performed as detailed previously (42).

### Quantitative real-time PCR

Quantitative real-time PCR was performed using total RNAs extracted from mutant (MT) and wild type (WT) fish using Totally RNA™ Kit (Ambion) and reverse transcribed with the PrimeScript™ RT Reagent Kit (Takara). FastStart Universal SYBR Green Masters (Roche) were utilized and the 2^–ΔΔCt^ method was used to normalize the genes of interest (Supplementary Table S2) to the endogenous housekeeping gene *gapdh*.

### Blue native electrophoresis analysis

Blue native gel electrophoresis (PAGE) was performed by isolating mitochondrial proteins from MT and WT zebrafish, as detailed previously (50). Samples containing 15 μg of proteins were separated on 3–11% Bis-Tris Native PAGE gel. The primary antibodies applied for this experiment were anti-Ndufa9 (Proteintech, 20312-1-AP), anti-Sdha (Proteintech, 14865-1-AP), anti-Uqcrc2 (Abcam, ab203832), anti-Cox5a (Proteintech, 11448-1-AP), anti-Atp5c (Proteintech 60284-1-Ig) and anti-Vdac1/2(Proteintech, 10866-1-AP). Peroxidase AffiniPure goat anti-mouse IgG and goat anti-rabbit IgG (Jackson) were used as secondary antibodies and protein signals were detected using the ECL system (CWBIO).

### Assays of activities of respiratory complexes

The enzymatic activities of Complexes I, II, III, IV and V were assayed as detailed elsewhere (51,52). Briefly, the activity of Complex I was determined through the oxidation of NADH with ubiquinone as the electron acceptor. Complex II was examined through the artificial electron acceptor DCPIP. The activity of Complex III was measured through the reduction of cytochrome *c* (III) by using d-ubiquinol-2 as the electron donor. The Complex IV was monitored through the oxidation of cytochrome *c* (II). The activity of complex V was explored through the NADH oxidation via conversion of phosphoenolpyruvate to lactate by two step reactions.

Enzyme histochemistry (EHC) staining for SDH and COX in the frozen-sections were performed as detailed elsewhere (53,54). Briefly, freshly dissected heart tissues were embedded in OCT compound (Tissue-Tek), frozen on dry ice, and sectioned to 10 μm. Tissue sections were then stained by SDH and COX for 15 min and 10 min, respectively.

### Whole mount *in situ* hybridization

Whole mount *in situ* hybridization was carried out as previously described (55–57). DIG-labeled antisense RNA probes specific to zebrafish *cmlc2* were synthesized from linearized pGEM-T Easy Vector, constructed by PCR‐amplifying fragments of the *cmlc2* cDNA spanning coding region (GenBank accession number: NM_131329). Zebrafish embryos from various age of post-fertilization were dechorionated in 2 mg/ml pronase in E3 medium and fixed at 4°C in 4% paraformaldehyde in phosphate-buffered saline (PBS) overnight, then transferred to 100% methanol for storage at −20°C overnight before undergoing hybridization. After the hybridization procedure, embryos were washed extensively in PBS with 0.1% Tween 20, re-fixed in 4% paraformaldehyde, and then transferred to 70% glycerol. Stained embryos were visualized using stereoscopic microscopes (SMZ18, Nikon) (42).

### Histological studies

For hematoxylin and eosin (H&E) staining, zebrafish was anesthetized, the heart was extracted and fixed in 10% formalin for 24 h at room temperature. Samples were then dehydrated, infiltrated, embedded in paraffin, sliced into 5 μm thick by pathologic microtome (RM2016, Leica). Tissue sections were then stained by hematoxylin and eosin (H&E) as described previously (58).

For wheat germ agglutinin (WGA) (Sigma) immunofluorescent staining, heart tissues were fixed in 4% paraformaldehyde at 4°C overnight, then washed with PBS and incubated in 30% sucrose solution at 4°C overnight. The samples were frozen in OCT compound at −80°C, and 10 μm cryo-sections were cut using a CM1950 cryostat (Leica). Frozen sections were washed in PBS for 10 min before incubated with FITC-conjugated WGA (1:50 in PBS, 1 mg/ml stock solution) for 1 h at room temperature. The nuclei were stained with DAPI (1:1000 in PBS, 10 μM stock solution, Sigma) for 10 mins at room temperature (59). Fluorescence was visualized with a confocal microscope (OLYMPUS, FV1000).

### Quantitative assessment of cardiac function

Offspring of the *gtpbp3* heterozygous *Tg* (*cmlc2*: *egfp*) were used for evaluation of cardiac function (60). The time-lapse image data analyses were performed as described previously (61,62). 5 dpf living larvae were transferred onto glass slides with drops of methylcellulose, and laid at the same orientation before videos were taken. The ventricles of the embedded larvae were observed under an epifluorescence microscope (SMZ25; Nikon, Tokyo, Japan), and images were recorded at 100 frames/s for 10 s. Time-lapse images, the long and short axis diastolic and systolic diameters of the ventricle were measured using Olympus software CellSense.

### Transmission electron microscopy

The ultrastructure of adult zebrafish hearts at 1 year old was observed using transmission electron microscopy. Heart-tissues were fixed in 2.5% glutaraldehyde, embedded in Epon 812, and cut into 100-nm thick slices using UC7 ultramicrotome (Leica, Heerbrugg, Switzerland), then stained with uranyl acetate and lead citrate. Finally, the images of myocardial ultra-structure were captured by a Hitachi-7650 transmission electron microscope (Hitachi, Tokyo, Japan).

### Computer analysis

Variance analysis was performed by the analysis of variance (ANOVA) test contained in GraphPad Prism 7 (Graphpad) and entering individual replicate values. Differences were considered significantly at a *P* < 0.05.

## RESULTS

### Generation of *gtpbp3* knock-out zebrafish using CRISPR/Cas9 system

**Figure 1.**
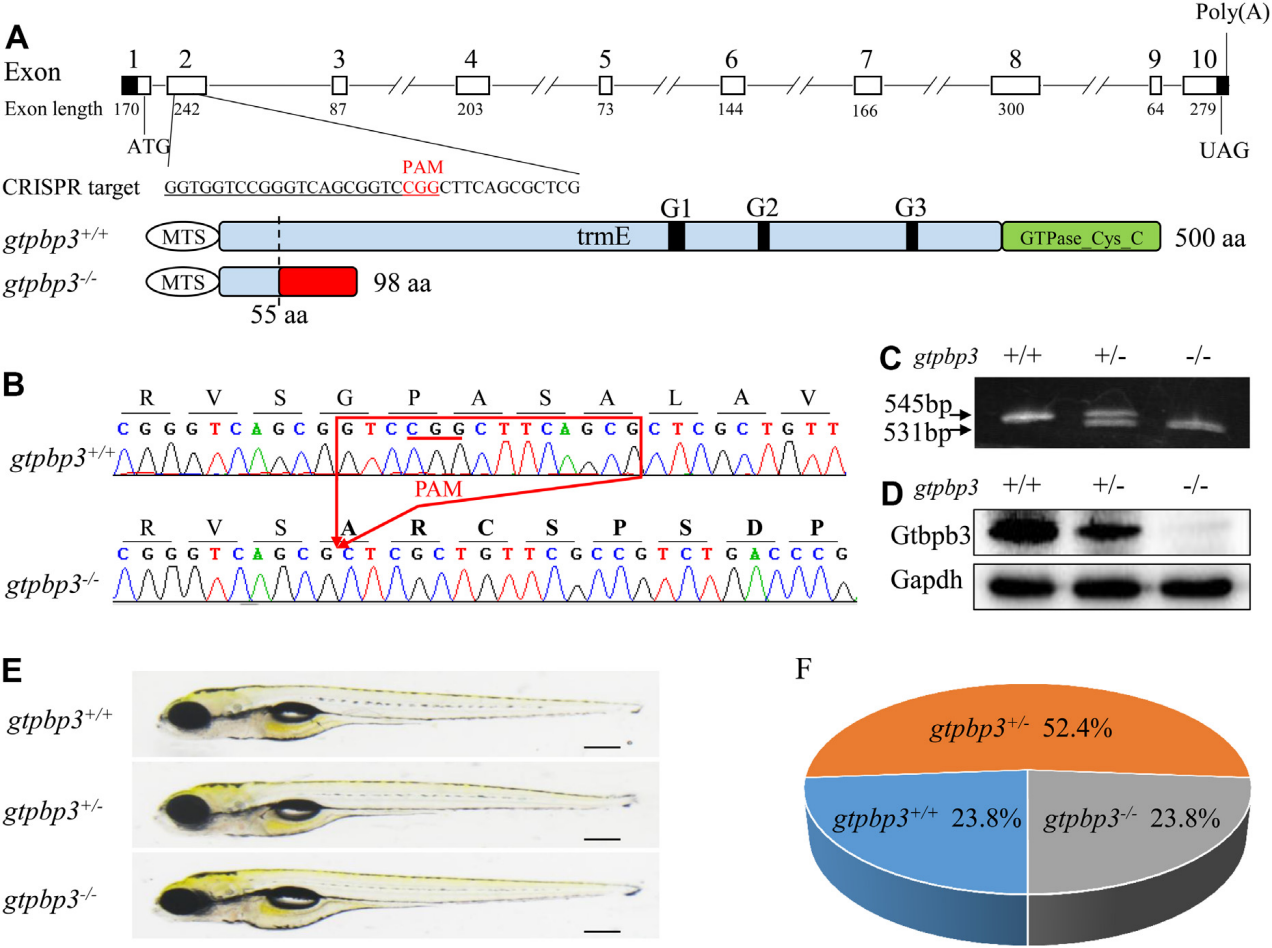
Genome editing of *gtpbp3* in zebrafish using CRISPR/Cas9 system. (**A**) Schematic representation of CRISPR/Cas9 target site at exon 2 of zebrafish *gtpbp3* gene as used in this study. White boxes indicate coding region, black boxes indicate untranslated regions of exons and lines between exons indicate introns. The resultant truncated 98 aa non-functional protein caused by frame shifting deletion in *gtpbp3* is shown in diagram. (**B**–**D**) Genotyping of *gtpbp3*^del14bp^ by Sanger sequence, the PAGE and Western blot analyses. (**E**) Lateral views of *gtpbp3*^+/+^, *gtpbp3*^+/−^, *gtpbp3*^−/−^ zebrafish at 5 dpf. (F) The ratios of genotypes/phenotype of offsprings (F2) in clutches from different F1 *gtpbp3*^+/-^ heterozygous crosses at 10 dpf (*n* = 42).

To investigate the pathological consequences of *gtpbp3* mutation, we used the CRISPR/Cas9 technology to generate zebrafish mutant lines where the Gtpbp3 ortholog was disrupted. As shown in Figure 1A, a single guide RNA (sgRNA) targeting exon 2 of *gtpbp3* was injected into wild-type one-cell stage embryos together with Cas9 mRNA. As a result, an allele, *gtpbp3^del14bp^* was generated by introducing a 14 bp deletion in the exon 2 of *gtpbp3* (heterozygous and homozygous zebrafish were described as *gtpbp3*^+/−^, *gtpbp3*^−/-^ and wild type as WT). In fact, this deletion caused a frameshift from codon 55 and the introduction of a premature stop at codon 98 (Figure 1A, B). This allele was subsequently propagated after confirmation of the mutation by Sanger sequencing, DNA-PAGE and Western blot analyses (Figure 1B–D). Both *gtpbp3*^+/−^ and *gtpbp3*^−/−^ zebrafish were adult-viable. Furthermore, there was no notable difference in gross morphology between mutant and wild type 5 dpf embryos (Figure 1E). The ratios of genotypes of offsprings (F2) in clutches from different F1 *gtpbp3* heterozygous crosses mirrored the Mendelian ratio (Figure 1F).

### Analysis of nucleotide modification and steady-state levels of mitochondrial tRNAs

**Figure 2.**
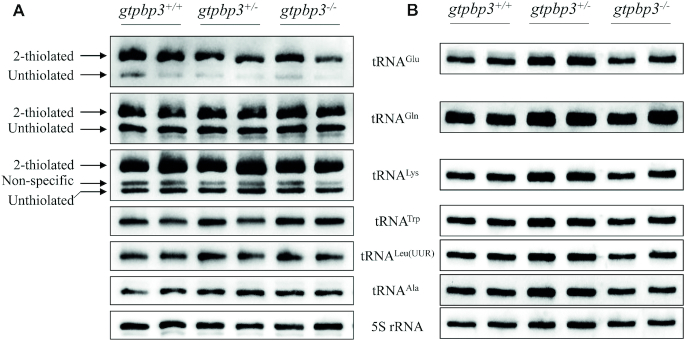
Northern blotting analysis of mitochondrial tRNAs. (**A**) APM gel electrophoresis. Five μg of mutant and WT zebrafish total RNAs were separated by polyacrylamide gel electrophoresis that contains 0.05 mg/ml APM, electroblotted onto a positively charged membrane, and hybridized with DIG-labeled oligonucleotide probes specific for tRNA^Glu^, tRNA^Gln^, tRNA^Lys^, tRNA^Trp^, tRNA^Leu(UUR)^, tRNA^Ala^ and 5S rRNA, respectively. The retarded bands of 2-thiolated tRNAs and non-retarded bands of tRNA without thiolation are marked by arrows. (**B**) Northern blot analysis of tRNA under a denaturing condition. Five μg of mutant and WT zebrafish total RNAs were electrophoresed through a denaturing polyacrylamide gel, electroblotted and hybridized with the same DIG-labeled oligonucleotide probes as described above.

It was hypothesized that the loss of Gtpbp3 led to a failure in tRNA metabolism (32). To investigate whether the loss of Gtpbp3 affected the nucleotide modification at position 34 of tRNAs in zebrafish, the 2-thiouridylation levels of tRNAs were determined by isolating total RNAs from *gtpbp3*^+/+^, *gtpbp3*^+/−^ and *gtpbp3*^−/−^ zebrafish, quantifying the 2-thiouridine modification by the retardation of electrophoresis mobility in APM polyacrylamide gel, and hybridizing DIG-labeled probes for mitochondrial tRNA^Glu^, tRNA^Gln^, tRNA^Lys^, tRNA^Trp^, tRNA^Leu(UUR)^ and tRNA^Ala^ as well as 5S rRNA as internal controls (40–42). In this system, the mercuric compound specifically interacted with the tRNAs containing the thiocarbonyl group, such as tRNA^Glu^, tRNA^Gln^ and tRNA^Lys^, thereby retarding tRNA migration. As shown in Figure 2A, 2-thiouridylation levels of mitochondrial tRNA^Glu^, tRNA^Gln^ and tRNA^Lys^ in *gtpbp3*^+/−^ and *gtpbp3*^−/−^ zebrafish were comparable with those in *gtpbp3*^+/+^ zebrafish.

To assess if the *gtpbp3* mutation altered the stability of tRNAs, we subjected total RNAs from mutant and WT zebrafish to Northern blotting and hybridized them with DIG-labeled oligodeoxynucleotide probes tRNA^Glu^, tRNA^Gln^, tRNA^Lys^, tRNA^Trp^, tRNA^Leu(UUR)^, tRNA^Ala^ and 5S rRNA, respectively (Figure 2B). For comparison, the levels of each tRNA were normalized to the reference 5S rRNA. The steady-state levels of tRNA^Lys^ was mildly reduced in *gtpbp3*^−/−^ zebrafish, but the levels of tRNA^Glu^, tRNA^Gln^, tRNA^Trp^, tRNA^Leu(UUR)^ and tRNA^Ala^ from *gtpbp3* mutant zebrafish were all comparable with those in WT zebrafish.

### Altered conformation of mitochondrial tRNAs

**Figure 3.**
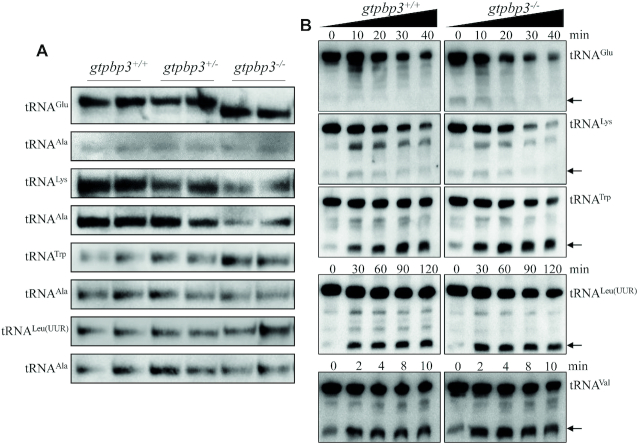
Analysis of mitochondrial tRNA conformation. (**A**) Northern blot analysis of tRNAs under native condition. Five μg of mutant and WT zebrafish total RNAs were electrophoresed through a native polyacrylamide gel, electroblotted and hybridized with the same DIG-labeled oligonucleotide probes as tRNA^Glu^, tRNA^Trp^, tRNA^Leu(UUR)^, tRNA^Lys^ and tRNA^Ala^, respectively. (**B**) S1 digestion patterns of tRNA^Glu^, tRNA^Trp^, tRNA^Leu(UUR)^, tRNA^Lys^ and tRNA^Val^, purified from *gtpbp3*^−/−^ and WT zebrafish. Two micrograms of RNAs were used for the S1 cleavage reaction at various lengths (from 0 to 120 min). Cleavage products of tRNAs were resolved in 10% denaturating PAGE gels with 8 M urea, electroblotted and hybridized with 3′ end DIG-labeled oligonucleotide probes specific for tRNA^Glu^, tRNA^Trp^, tRNA^Leu(UUR)^, tRNA^Lys^ and tRNA^Val^, respectively. Arrows denoted the 30 to 40 nt regions showing 3′ tRNA half.

Modifications of tRNA participate in forming the structure of a certain tRNA (63–64). It is anticipated that the deficient nucleotide modification may affect the conformation of mitochondrial tRNA. To test this hypothesis, total RNAs were electrophoresed through 10% polyacrylamide gel (native condition) in Tris-glycine buffer and then electroblotted onto a positively charged nylon membrane for hybridization analysis with DIG-labeled oligodeoxynucleotide probes for tRNA^Glu^, tRNA^Lys^, tRNA^Trp^, tRNA^Leu(UUR)^ with taurine modification and tRNA^Ala^ without taurine modification (14), respectively. As shown in Figure 3A, electrophoretic patterns under native condition showed that the tRNA^Glu^, tRNA^Lys^, tRNA^Trp^ in *gtpbp3*^−/−^ zebrafish migrated faster than those of WT zebrafish, while tRNA^Leu(UUR)^ in *gtpbp3*^−/−^ zebrafish migrated slightly slower than those in WT zebrafish. However, there were no obvious electrophoretic mobility differences of the tRNA^Ala^ between *gtpbp3*^−/−^ and WT zebrafish.

We further evaluated whether the loss of Gtpbp3 perturbed the structures of tRNAs by analyzing the sensitivity of tRNA^Glu^, tRNA^Lys^, tRNA^Trp^ and tRNA^Leu(UUR)^ (present taurine modification in WT) as well as tRNA^Val^ (absent taurine in WT), from *gtpbp3*^−/−^ and WT zebrafish to digestion with the nuclease S1. The resultantly digested-products from *gtpbp3*^−/−^ and WT zebrafish were then followed by Northern blot analysis using tRNA probes that hybridized only to 3′ half tRNAs. As illustrated in Figure 3B, the tRNA^Glu^, tRNA^Lys^, tRNA^Trp^ and tRNA^Leu(UUR)^ from *gtpbp3*^−/−^ zebrafish were more sensitive to S1-mediated digestion than those from WT zebrafish and exhibited remarkable differences in S1-mediated digestion patterns of tRNAs from *gtpbp3*^−/−^ zebrafish. Notably, tRNA^Glu^ was the most sensitive to S1 digestion, even the tRNA half of tRNA^Glu^ was already present only in *gtpbp3*^−/−^ zebrafish. The sensitive tRNA^Glu^ from *gtpbp3*^−/−^ zebrafish to S1-mediated digestion was correlated with the drastic changes of electrophoretic mobility. Furthermore, the nuclease S1 degraded full-length tRNA^Lys^, tRNA^Leu(UUR)^ and tRNA^Trp^ faster in *gtpbp3*^−/−^ zebrafish than those in WT. As shown in Supplemental Figure S3, the ratios of tRNA half/full length tRNA^Glu^, tRNA^Lys^ and tRNA^Leu(UUR)^ did not differ significantly between *gtpbp3*^−/−^ and WT zebrafish, respectively, whereas the ratio of tRNA half/full-length tRNA^Trp^ in *gtpbp3*^−/−^ zebrafish increased significantly more than those in WT zebrafish during the digestion time. Conversely, there was no significant difference between the sensitivity of tRNA^Val^ from *gtpbp3*^−/−^ and WT zebrafish to digestion with the nuclease S1. These data validated that the inactivation of Gtpbp3 changed the conformation of mitochondrial tRNAs.

### Increased aminoacylation of mitochondrial tRNAs

**Figure 4.**
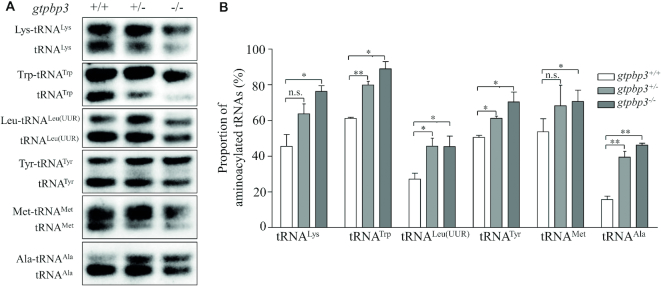
*In vivo* aminoacylation assays. (**A**) Five μg of *gtpbp3*^+/−^ and *gtpbp3*^−/−^ and WT zebrafish total RNAs under acid conditions were electrophoresed at 4°C through an acid (pH 5.2) 10% polyacrylamide with 8 M urea gel, electroblotted, and hybridized with a DIG-labeled oligonucleotide probes specific for the tRNA^Trp^, tRNA^Lys^, tRNA^Leu(UUR)^, tRNA^Tyr^, tRNA^Ala^ and tRNA^Met^, respectively. (**B**) Quantification of aminoacylated proportions of tRNAs in mutant and WT zebrafish. The calculations were based on three independent experiments. The error bars indicate two standard deviations (SD) of the means. *P* indicates the significance, according to ANOVA test, of the difference between *gtpbp3*^+/−^ or *gtpbp3*^−/−^ and WT values for proteins, denoted by asterisks (*P< 0.05, **P< 0.01, ***P< 0.001), and non-significant differences by n.s.

To assess whether the loss of Gtpbp3 affected the aminoacylation of tRNA, we examined the aminoacylation levels of tRNA^Lys^, tRNA^Leu(UUR)^, tRNA^Trp^, tRNA^Tyr^, tRNA^Ala^ and tRNA^Met^ by using electrophoresis in an acidic urea PAGE system to separate uncharged tRNA species from the corresponding charged tRNA, electroblotting and hybridizing with the tRNA probes described above. As shown in Figure 4A, the tRNA^Lys^, tRNA^Leu(UUR)^ and tRNA^Trp^ in the *gtpbp3*^−/−^ zebrafish migrated slightly faster than those of WT zebrafish, while there were no obvious differences in electrophoretic mobility of tRNA^Tyr^, tRNA^Ala^ and tRNA^Met^ between *gtpbp3*^−/−^ and WT zebrafish. Notably, *gtpbp3*^−/−^ and *gtpbp3*^+/-^ zebrafish exhibited marked increases in the aminoacylated levels of mitochondrial tRNAs, as compared with those in WT zebrafish. As shown in Figure 4B and Table 1, the efficiencies of aminoacylated tRNA^Lys^, tRNA^Leu(UUR)^, tRNA^Trp^, tRNA^Tyr^, tRNA^Ala^ and tRNA^Met^ in the *gtpbp3*^−/−^ zebrafish were 168%, 167%, 146%, 140%, 295% and 132% of those in WT zebrafish, the efficiencies of aminoacylated tRNA^Lys^, tRNA^Leu(UUR)^, tRNA^Trp^, tRNA^Tyr^, tRNA^Ala^ and tRNA^Met^ in the *gtpbp3*^+/−^ zebrafish were 140%, 168%, 131%, 121%, 252% and 127% of those in WT zebrafish.

**Table 1.** Quantification of the levels of aminoacylated mitochondrial tRNAs (%)

	tRNA^Lys^	tRNA^Trp^	tRNA^Leu(UUR)^	tRNA^Tyr^	tRNA^Met^	tRNA^Ala^
*gtpbp3* ^+/+^	45 ± 5	58 ± 3	27 ± 2	48 ± 2	54 ± 5	16 ± 1
*gtpbp3* ^+/−^	64 ± 4	76 ± 5	46 ± 3	58 ± 2	68 ± 8	39 ± 2
*gtpbp3* ^−/−^	76 ± 2	85 ± 7	45 ± 4	67 ± 7	71 ± 4	46 ± 1

The average levels of aminoacylated mitochondrial tRNA^Lys^, tRNA^Trp^, tRNA^Leu(UUR)^, tRNA^Tyr^, tRNA^Met^ and tRNA^Ala^were based on three independent determinations of each tRNA in mutant and wild type zebrafish. The values were expressed as percentages of the average values for the aminoacylated mitochondrial tRNA.

### Reductions in the levels of mitochondrial proteins

**Figure 5.**
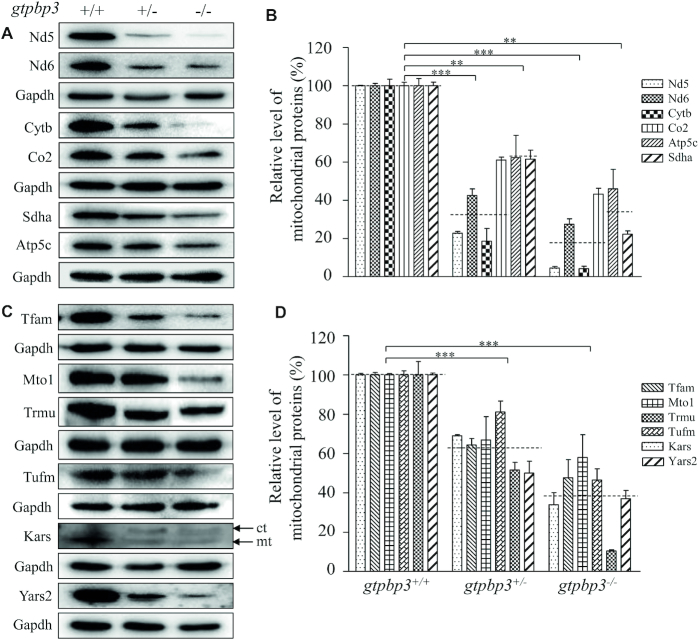
Western blotting analysis of mitochondrial proteins. (**A, C**) Twenty micrograms of total proteins from mutant and WT zebrafish at the age of one year were electrophoresed through a denaturing polyacrylamide gel, electroblotted and hybridized with antibodies for 6 subunits of OXPHOS (four encoded by mtDNA and two encoded by nuclear genes), Tfam, Mto1, Trmu, Kars, Yars2, Tufm as well as Gapdh as a loading control. Quantification of levels of OXPHOS subunits (**B**) and other mitochondrial proteins (**D**). Average content of Nd5, Nd6, Cytb, Co2, Atp5c, Sdha, Tfam, Mto1, Trmu, Kars, Yars2 and Tufm was normalized to the average content of Gapdh in mutant and wild type zebrafish. The horizontal dashed lines represent the average values of four proteins encoded by mtDNA or two proteins encoded by nuclear genes for each group (B). The calculations were based on three independent determinations. Graph details and symbols are explained in the legend to Figure 4.

To investigate whether the aberrant mitochondrial tRNA metabolism altered mitochondrial translation, Western blotting analysis was carried out to examine the subunits of OXPHOS, including four mtDNA encoding polypeptides (Nd5, Nd6, Cytb and Co2), two nuclear encoding proteins Atp5c and Sdha and four mitochondrial proteins: non-OXPHOS subunits (Kars, Yars2, Tufm and Tfam) in mutant and WT zebrafish using Gapdh as a loading control. As shown in Figure 5A, the levels of Nd5, Nd6 (subunits 5 and 6 of NADH dehydrogenase), Cytb (subunit of ubiquinol-cytochrome c reductase), Co2 (subunit II of cytochrome c oxidase), Sdha (subunit of succinate dehydrogenase), Atp5c (subunit of H^+^-ATPase), Tfam (transcription factor A, mitochondrial), Mto1 (mitochondrial tRNA translation optimization 1), Trmu (tRNA 5-methylaminomethyl-2-thiouridylate methyltransferase), Tufm (Tu translation elongation factor, mitochondrial), Kars (lysyl-tRNA synthetase) and Yars2 (tyrosyl-tRNA synthetase 2) were decreased in mutant zebrafish, as compared with those in WT zebrafish. Notably, the levels of Nd5, Nd6, Cytb and Co2 in *gtpbp3*^−/−^ zebrafish were 5%, 27%, 4% and 43%, with the average of 18%, relative to the mean values measured in the WT zebrafish (*P* < 0.0001). Furthermore, the average levels of these mtDNA-encoding proteins in *gtpbp3*^+/−^ zebrafish were 36%, relative to the average values in WT zebrafish. Moreover, the levels of Atp5a and Sdha in the *gtpbp3*^−/−^ zebrafish were 46% and 22% of the average values measured in the WT zebrafish (Figure 5B). As showed in Figure 5D, the levels of Tfam, Mto1, Trmu, Tufm, Kars and Yars2 in *gtpbp3*^−/−^ zebrafish were 34%, 48%, 58%, 47%, 10% and 37%, relative to the mean value measured in WT zebrafish (*P* < 0.05).

### Imbalances in protein stoichiometry of OXPHOS complexes

To determine if these decreased nuclear-encoding proteins resulted from transcriptional downregulation, we performed the real-time PCR analysis of *sdha, atp5c, tfam, mto1, trmu, tufm, kars and yars2*. As shown in Supplemental Figure S4, no significant differences in the levels of these eight genes were observed among the MT and WT zebrafish.

**Figure 6.**
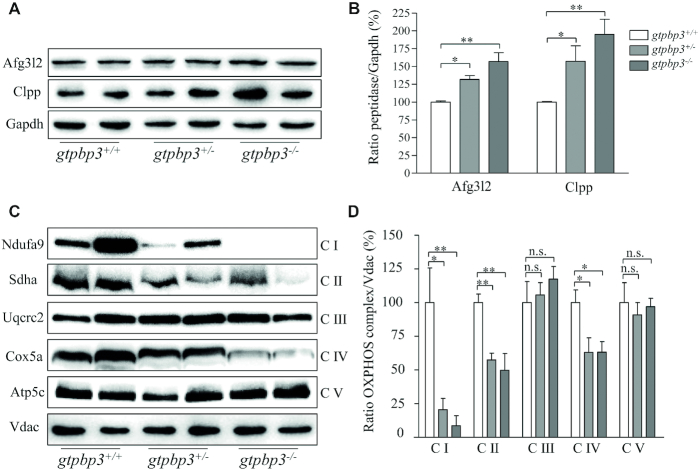
Imbalances in protein stoichiometry of OXPHOS complexes. (**A**) Western blotting analysis of Afg3l2 and Clpp proteins. Twenty micrograms of total cellular proteins from mutant and WT zebrafish were electrophoresed through a denaturing polyacrylamide gel, electroblotted and hybridized with antibodies for Afg3l2 and Clpp as well as Gapdh as a loading control. (**B**) Quantification of levels of Afg3l2 and Clpp. The calculations were based on three independent experiments. (**C**) The steady-state levels of five OXPHOS complexes by Blue-Native gel electrophoresis. Fifteen μg of mitochondrial proteins from mutant and WT zebrafish were electrophoresed through a Blue-Native gel, electroblotted and hybridized with antibodies for Ndufa9, Sdha, Uqcrc2, Cox5a, Atp5c (subunits of complex I, II, III, IV and V, respectively) as well as Vdac as a loading control. (**D**) Quantification of levels of complexes I, II, III, IV and V in mutant and WT zebrafish. The calculations were based on three independent experiments. Graph details and symbols are explained in the legend to Figure 4.

To test whether the Gtpbp3-defiency affected the mitochondrial proteostasis, we measured the levels of Clpp involved in mitochondrial ribosome assembly (65) and ATP family gene 3-like2 (Afg3l2) proteases involved in the turnover of misfolded proteins markers for proteostasis stress (66), in the MT and WT zebrafish. As shown in Figure 6A,B, the levels of Afg3l2 and Clpp in *gtpbp3*^−/−^ zebrafish were 157% and 195%, and the levels of Afg3l2 and Clpp in *gtpbp3*^+/−^ zebrafish were 132% and 157%, relative to the mean values measured in the WT zebrafish, respectively. These data indicated that *gtpbp3* mutation produced the proteostasis stress.

To investigate whether the *gtpbp3* mutation-induced alterations affected the stability of OXPHOS complexes, we measured the steady-state levels of five OXPHOS complexes by Blue-Native gel electrophoresis. As shown in Figure 6C,D, the levels of complex I (C I), complex II (C II) and complex IV (C IV) in *gtpbp3*^−/−^ zebrafish were 9%, 50% and 63% of the mean values measured in the WT zebrafish, respectively. Similarly, the levels of C I, C II and C IV in *gtpbp3*^+/−^ zebrafish were 20%, 57% and 63%, relative to the mean values measured in WT zebrafish. However, the levels of C III and C V in *gtpbp3*^−/-^ and *gtpbp3*^+/−^ were comparable with those in WT zebrafish. The lower levels of the respiratory complexes I, II and IV may be due to the misfolded and/or misassembled.

### Reduced activities of respiratory complexes

**Figure 7.**
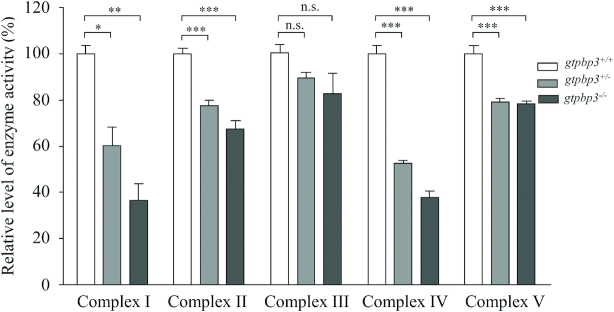
Enzymatic activities of respiratory chain complexes. The activities of respiratory complexes were investigated by enzymatic assays on complexes I, II, III, IV and V in mitochondria isolated from mutant and wild type zebrafish at the ages of one year. The calculations were based on three independent determinations. Graph details and symbols are explained in the legend to Figure 4.

To examine the effect of *gtpbp3* mutation on the oxidative phosphorylation, we measured the activities of respiratory complexes by isolating mitochondria from *gtpbp3*^+/+^, *gtpbp3*^+/−^ and *gtpbp3*^−/−^ zebrafish. Complex I activity was determined by following the oxidation of NADH with ubiquinone as the electron acceptor (51,52). The activity of complex II (succinate ubiquinone oxidoreductase), which was exclusively encoded by the nuclear DNA, was examined by the artificial electron acceptor DCPIP. Complex III (ubiquinone cytochrome c oxidoreductase) activity was measured as the reduction of cytochrome *c* (III) using d-ubiquinol-2 as the electron donor. Complex IV (cytochrome c oxidase) activity was monitored by following the oxidation of cytochrome *c* (II). Complex V (F1, F0-ATP synthetase) activity was measured as the oxidation of NADH using phosphoenolpyruvate as the electron acceptor (51,67,68). As shown in Figure 7, the activities of Complex I, II, III, IV and V in *gtpbp3*^−/−^ zebrafish were 36.5%, 67.5%, 82.7%, 37.8% and 78.3%, relative to the mean values measured in the WT zebrafish, respectively. Furthermore, the activities of Complex I, II, III, IV and V in *gtpbp3*^+/-^ zebrafish were 60.4%, 77.4%, 89.4%, 52.8% and 79.2% of the average values measured in WT zebrafish, respectively.

### Loss of Gtpbp3 altered embryonic heart development

**Figure 8.**
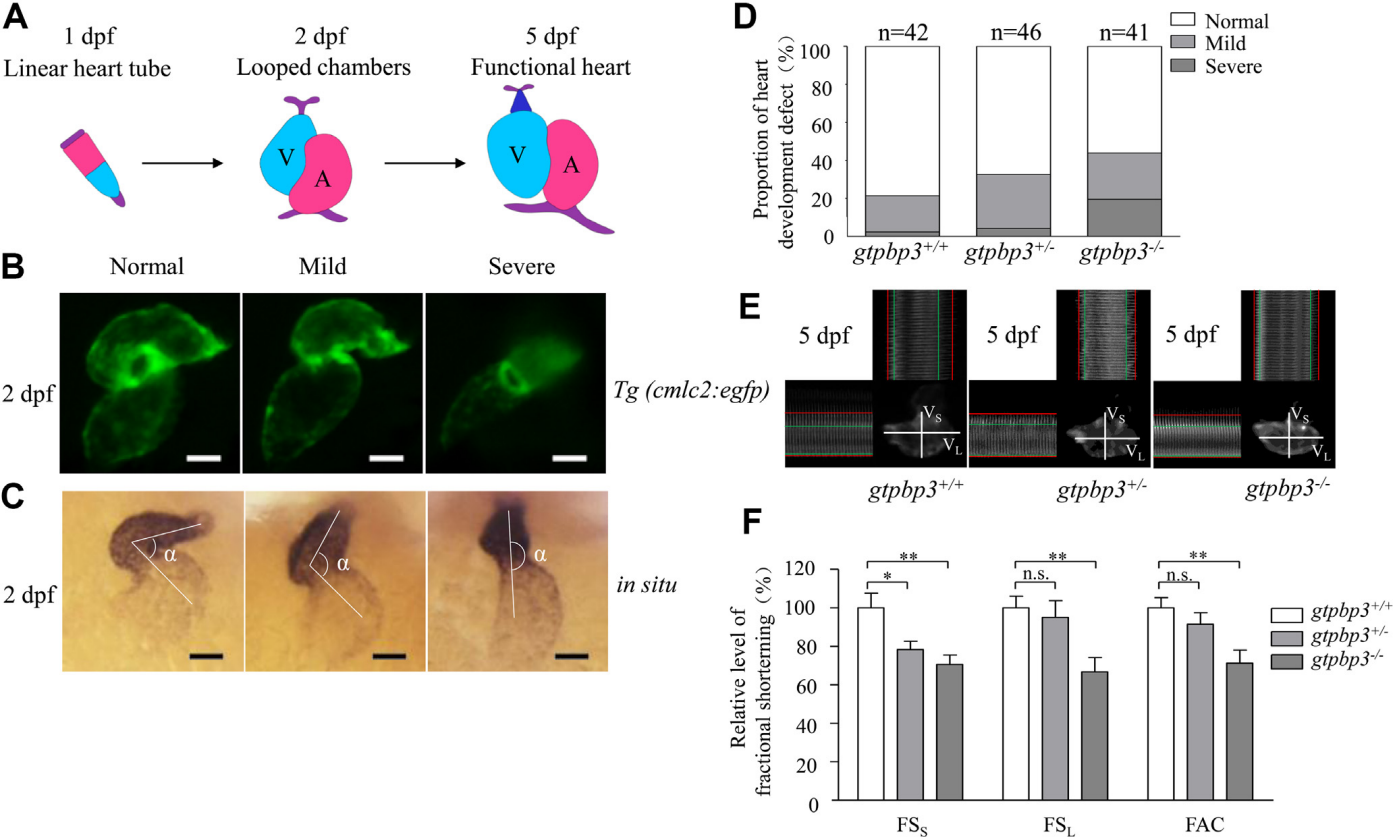
Cardiac defects in zebrafish. (**A**) Schematic diagram of zebrafish heart development at 1, 2 and 5 dpf. V, ventricle; A, atrium. (**B**) *Tg (cmlc2:egfp)* embryos at 2 dpf shows heart-restricted GFP expression in both chambers. (**C**) Whole-mount *in situ* hybridization against *cmlc2*. According to the looping state, measured by the angles between ventricle and atrium, hearts were divided into normal (α<90°), mild (90°<α<180°), and severe (α > 180°). (**D**) The proportions of each phenotypes in the embryos in mutant and WT zebrafish. (**E**) Time-lapse images, the ventricular long axis and ventricular short axis were labeled as V_L_ and V_S_, respectively. End diastolic (red line) and systolic (green line) diameters of zebrafish ventricle at 5 dpf were measured using Olympus software CellSense. (**F**) The proportions of the short axis fractional shortening (FS_S_), long axis fractional shortening (FS_L_) and fractional area change (FAC) in mutant and WT zebrafish. the calculations were based on eight independent determinations. Graph details and symbols are explained in the legend to Figure 4.

Heart was the first organ to form and function during zebrafish embryonic development (69–71). As shown in Figure 8A, cardiac primordium went through migration and left-jogging process within the first day post fertilization and initiated contraction around 1 dpf. The chambers of ventricle and atrium formed and the whole heart underwent an S-looping process at 2 dpf. Finally, the functional heart was formed at 5 dpf (72,73). Cardiac-specific marker *cmlc2* was used to investigate the effect of *gtpbp3* deletion on heart development by either green fluorescence or whole-mount *in situ* hybridization at 2 dpf. As shown in Figure 8B, *gtpbp3*^ko^ zebrafish exhibited various degrees of perturbed S-looping processing. Based on looping angles between ventricle and atrium, zebrafish hearts were divided into categories of normal (α<90°), mild (90° < α < 180°), and severe (α>180°) (Figure 8C). As shown in Figure 8D, 19% *gtpbp3*^+/+^ (*n* = 42), 28% *gtpbp3*^+/−^ (*n* = 46) and 24% *gtpbp3*^−/–^ (*n* = 41) displayed mild-loop heart, respectively. In contrast, 2% *gtpbp3*^+/+^ (*n* = 42), 4% *gtpbp3*^+/−^ (*n* = 46) and 20% *gtpbp3*^−/–^ (*n* = 41) had no-loop heart, respectively. These data indicated that the deletion of *gtpbp3* led to defects in embryonic heart development.

We then evaluated if the loss of Gtpbp3 altered the heart function of zebrafish at 5dpf by time-lapse images. This technique allowed us to examine the extent of ventricle during the process of diastole and systole. As illustrated in Figure 8E, ventricular functions were evaluated by measuring the fractional shortening level of short axis (FS_S_), fractional shortening level of long axis (FS_L_) and fractional area change (FAC) of ventricle. As shown 8F, FS_S_, FS_L_ and FAC of *gtpbp3*^+/-^ zebrafish were 78% (*P* = 0.0153), 95% (*P* = 0.6856) and 91% (*P* = 0.3386) of the mean values measured in WT zebrafish, respectively, while FS_S_, FS_L_ and FAC of *gtpbp3*^−/-^ zebrafish were 71% (*P* = 0.0038), 67% (*P* = 0.0039) and 71% (*P* = 0.0056) of the mean values measured in WT zebrafish, respectively. These results demonstrated that the deletion of *gtpbp3* altered cardiac function.

### The *gtpbp3* mutants exhibited hypertrophic cardiomyopathy in adult zebrafish.

**Figure 9.**
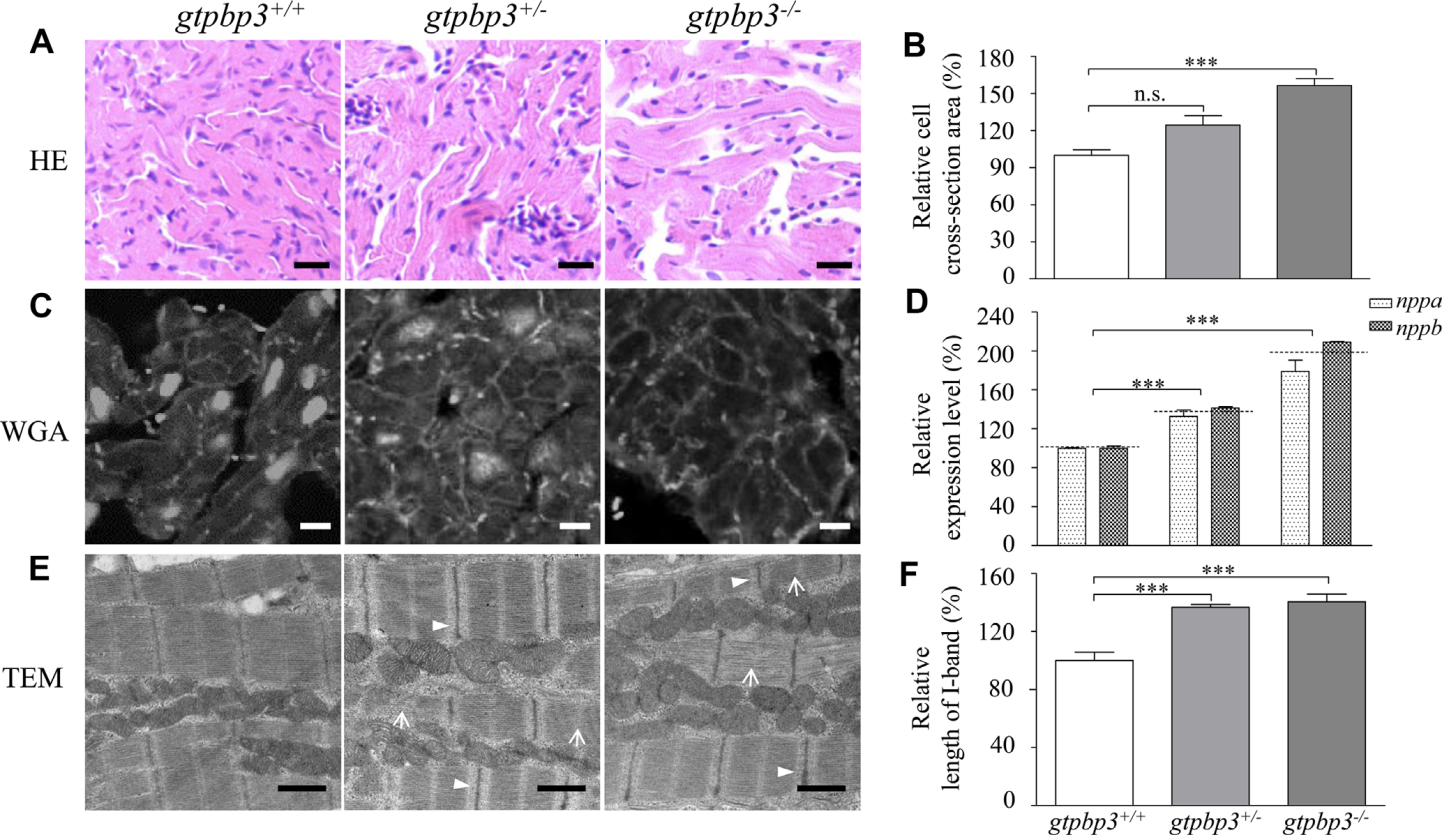
Hypertrophic cardiomyopathy in zebrafish. (**A**) Hematoxylin and eosin stained (H&E) histological sections of hearts from *gtpbp3*^−/−^, *gtpbp3*^+/−^ and *gtpbp3^+/+^* zebrafish at the age of one year. Scale bars: 10 μm. (**B**) The relative cross-sectional area of cardiomyocyte in *gtpbp3*^+/+^ (*n* = 136), *gtpbp3*^+/-^ (*n* = 147), *gtpbp3*^−/−^ (*n* = 116) zebrafish, staining with H&E. (**C**) The cardiomyocytes from mutant and WT zebrafish were visualized via WGA staining. Scale bars: 5 μm. (**D**) Relative levels of *nppa* and *nppb* gene in hearts from mutant and WT zebrafish. (**E**) Ventricular heart muscle sections of transmission electron microscopy in mutant and WT zebrafish. Ultrathin sections were visualized with 15,000X magnifications. Altered myofibrils were indicated by white arrows and widened I-band in the sarcomere units were indicated by white arrowhead. Scale bars: 1 μm. (**F**) Quantification of the I-band length of *gtpbp3*^+/+^ (*n* = 16), *gtpbp3*^+/-^ (*n* = 38) and *gtpbp3*^−/−^ (*n* = 37) zebrafish. The values for the mutants were expressed as percentages of the average values for the wild type.

We then investigated if the *gtpbp3*^ko^ zebrafish recapitulated the hypertrophic cardiomyopathy phenotypes in patients carrying the *GTPBP3* mutations. As shown in Figure 9A, cardiomyocytes of one-year-old *gtpbp3*^−/−^ zebrafish stained with hematoxylin/eosin (H&E) showed hypertrophy of cardiac myocytes and myocardial fiber disarray. The abnormal myocytes included the enlarged size, bizarre-shaped nuclei and disorganized patterns. Furthermore, the volumes of the interstitial matrix were increased in the *gtpbp3*^−/−^ zebrafish. As shown in Figure 9B, the cross-sectional areas of cardiomyocytes in *gtpbp3*^−/−^ and *gtpbp3*^+/-^ zebrafish were 156% (*P* = 0.0007) and 124% (*P* = 0.0546) relative to the mean values measured in WT zebrafish. As illustrated in Figure 9C, D, these phenotypes were verified by direct staining the outline of cardiomyocyte by FITC-conjugated WGA and marked increasing expression of *nppa* and *nppb* genes (HCM marker) in *gtpbp3^−/−^ and gtpbp3^+/-^ zebrafish* (74–76). As shown in Figure 9E, the *gtpbp3*^+/−^ and *gtpbp3*^−/−^ zebrafish exhibited defects in cardiac myofibrils and widened I-bands using transmission electron micrographs. As shown in Figure 9F, the lengths of I-bands were significantly increased *in gtpbp3^−/−^ and gtpbp3^+/−^*zebrafish, as compared with those in WT zebrafish. These data demonstrated that *gtpbp3^−/−^*zebrafish recapitulated the clinical phenotypes in HCM patients carrying the *GTPBP3* mutations.

### Mitochondrial defects in cardiomyocytes

**Figure 10.**
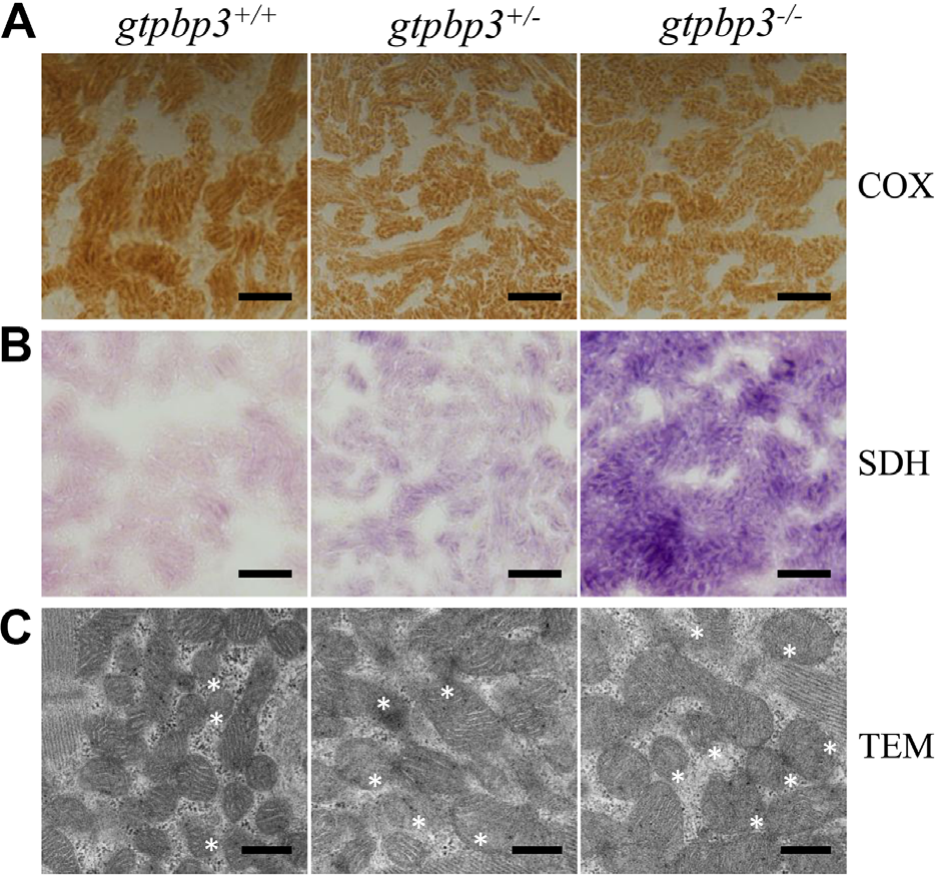
Mitochondrial defects in the zebrafish heart. (**A, B**) Assessment of mitochondrial function in cardiomyocytes by enzyme histochemistry (EHC) staining for COX (A) and SDH (B) in the frozen-sections of ventricles in the *gtbpbp3*^−/−^, *gtpbp3*^+/−^ and *gtpbp3*^+/+^ zebrafish at one year old. Scale bars: 40 μm. (C) Mitochondrial networks from cardiomyocytes of transmission electron microscopy. Ultrathin sections were visualized with 30 000× magnifications.

Mitochondrial dysfunction in cardiomyocytes was assessed by enzyme histochemistry staining for SDH and COX in the frozen-sections of ventricles of *gtpbp3^−/−^, gtpbp3^+/-^*and WT zebrafish at one year old. As shown in Figure 10A and B, mildly reduced activity of COX, but significantly increased activity of SDH were observed in *gtpbp3^−/−^* zebrafish, as compared to the wild type zebrafish. Mitochondrial defects in cardiomyocytes of mutant and WT zebrafish at one year old were further evaluated by using transmission electron microscope. As shown in Figure 10C, cardiomyocytes of *gtpbp3^−/−^* mutant zebrafish exhibited abnormal mitochondrial morphology including enlarged mitochondria and the partial loss of cristae, as compared to those of wild type zebrafish. Especially, mitochondrial volumes in *gtpbp3^−/−^* (*n* = 92) and *gtpbp3^+/−^* (*n* = 65) mutant cells were 176% and 172%, related to the average values of control cells (*n* = 147). The huge volume mitochondria were probably due to compensation for mitochondrial dysfunction.

## DISCUSSION

In the present study, we investigated the impacts of Gtpbp3 in mitochondrial biogenesis and cardiac function by characterizing *gtpbp3* knockout zebrafish generated using CRISPR/Cas9 system. Gtpbp3 is a highly conserved tRNA modifying enzyme responsible for the biogenesis of τm^5^U at the wobble position of mitochondrial tRNA^Glu^, tRNA^Gln^, tRNA^Lys^, tRNA^Trp^ and tRNA^Leu(UUR)^ (11,14,22,36,40,77,78). These were further evidenced by the complete loss of τm^5^U at the wobble position of mitochondrial tRNA^Glu^, tRNA^Gln^, tRNA^Lys^, tRNA^Trp^ and tRNA^Leu(UUR)^ in the human *GTPBP3* KO cells (14). It was hypothesized that the *gtpbp3* deletion resulted in the aberrant structure and function of tRNAs including the stability, functional folding and aminoacylation. However, the inactivation of *gtpbp3* did not affect the 2-thiouridylated levels at U34 of tRNA^Lys^, tRNA^Glu^ and tRNA^Gln^ in zebrafish, in contrast with the complete loss of τm^5^s^2^U at U34 of tRNA^Lys^, tRNA^Glu^ and tRNA^Gln^ in Trmu-KO zebrafish (42). These data further supported that the thiolation at position 2 was independent of the modification at position 5 (14,79). Furthermore, the loss of Gtpbp3 caused only mildly reduced level of tRNA^Lys^ but did not change the levels of other five tRNAs in the *gtpbp3*^−/−^ zebrafish. The inactivation of Gtpbp3 led to different effects of tRNA^Glu^, tRNA^Lys^, tRNA^Trp^ and tRNA^Leu(UUR)^ on the tertiary structure including functional folding of tRNAs. These were evidenced by various electrophoretic mobility changes and sensitivity to S1-mediated digestion of these tRNA in the *gtpbp3*^−/−^ zebrafish, as compared with those in WT zebrafish. These different effects are likely due to the different types of modifications at U34 in mitochondrial tRNA^Glu^, tRNA^Gln^, tRNA^Lys^, tRNA^Trp^ and tRNA^Leu(UUR)^, even though these tRNAs shared identical taurine modification at U34 (4, 14). Strikingly, the loss of Gtbpb3 indeed increased the aminoacylated levels of tRNA^Lys^, tRNA^Trp^, tRNA^Leu(UUR)^, tRNA^Tyr^, tRNA^Met^ and tRNA^Ala^ in the mutant zebrafish. The elevated levels of aminoacylated tRNAs in mutant zebrafish may be due to some levels of stabilization by compensatory effects (18,44,80). Alternatively, the unmodified U34 of these tRNAs may lead to mitochondrial ribosome pausing at their cognate codon and make the accumulation of aminoacylated tRNAs (81). Moreover, the increasing aminoacylated levels of these tRNAs may also result from the elevation of misacylated tRNAs caused by defective nucleotide modifications (82). The deficient modifications at wobble position of tRNAs caused by the *gtpbp3* deletion may affect the fidelity and efficiency of mitochondrial translation. In fact, the loss of τm^5^U modification may destabilize the U:G wobble base-pairing by increasing stacking interactions and affect the decoding accuracy through altered codon-anticodon interactions (6,23,83,84). Alternatively, the deficient τm^5^U modifications caused the inefficiency of mitochondrial translation (82,85,86). In the present study, 66% and 82% decreases in the average levels of 4 mtDNA encoding proteins were observed in the *gtpbp3*^+/−^ and *gtpbp3*^−/−^ mutant zebrafish, respectively. These results were consistent with the reduced rates of mitochondrial translation observed in human cell lines carrying the *GTPBP3* mutations or depletions (32,35).

The mitochondrial translational defects led to the imbalances between the increased levels of *de novo* protein synthesis and decreased folding capacity for the mtDNA- and nucleus-encoded mitochondrial proteins (87). Hence, the *gtpbp3* mutation-induced translational defects may lead to a buildup of unfolded and/or unassembled subunits of OXPHOS, thereby reducing the steady-state levels of OXPHOS complexes. The aberrant mitochondrial translation produced the proteostasis stress, such as increased protein degradation or misfolding in mitochondria, was evidenced by the increased levels of Afg3l2 and Clpp in *gtpbp3*^−/-^ and *gtpbp3*^+/−^ zebrafish (65,66). As a result, these alterations resulted in various reductions in nuclear encoding mitochondrial proteins:Atp5c, Sdha, Tfam, Mto1, Trmu, Tufm, Kars and Yars2. The defective mitochondrial translations resulted in the defective activities of complex I, II, III, IV and V, deficient ATP synthesis, and subsequent failure of cellular energetic process (88,89). These mitochondrial dysfunctions were further verified by the altered activities of SDH and COX and abnormal mitochondrial morphology in the cardiomyocytes of *gtpbp3*^−/−^ mutant zebrafish. These data demonstrated that the *gtpbp3* knock-out zebrafish recapitulated the biochemical phenotypes in human cell lines carrying the mutations and knockdown of *GTPBP3* gene (14,32,35,90).

The heart is the most energy-demanding organ in vertebrate body. Therefore, we hypothesized that mitochondrial dysfunction caused by Gtpbp3-deficieny particularly ablated the heart function. In zebrafish, cardiac primordium went through migration and left-jogging process around 1 dpf, the chambers of ventricle, and atrium formed and the whole heart underwent an S-looping process at 2 dpf and the functional heart was formed at 5 dpf (73–75). In this study, the *gtpbp3^−/−^* mutant larvae at 2 dpf revealed the abnormal S-loops in heart, indicating that the loss of Gtpbp3 altered the embryonic heart development. The cardiac defects were further evidenced by the fact that the *gtpbp3^−/−^* mutant zebrafish exhibited the reduced fractional shortening of ventricles at 5dpf. Furthermore, *gtpbp3^−/−^* adult zebrafish displayed the hypertrophy of cardiac myocytes and myocardial fiber disarray in ventricles. These cardiac defects in *gtpbp3^−/-^*zebrafish recapitulated the clinical phenotypes in HCM patients carrying the *GTPBP3* mutations (32,91–93). These data demonstrated that the *gtpbp3*-deletion-induced mitochondrial dysfunctions caused hypertrophic cardiomyopathy. Thus, our findings may provide new insights into the pathophysiology of hypertrophic cardiomyopathy, which was manifested by the aberrant nucleotide modification of mitochondrial tRNAs.

In summary, our findings demonstrated the pathophysiology of hypertrophic cardiomyopathy underlying GTPBP3 deficiency using the *gtpbp3* knock-out zebrafish. The deficient modifications caused by the deletion of Gtpbp3 perturbed the tertiary structures and function of mitochondrial tRNA. The aberrant mitochondrial tRNA metabolisms produced the impairment of mitochondrial translation, proteostasis, instability of OXPHOS, respiratory phenotypes and decreased ATP production. These mitochondria dysfunctions caused the alterations in the embryonic heart development, the reduced fractional shortening of ventricles, hypertrophy of cardiac myocytes and myocardial fiber disarray in ventricles. These cardiac defects in *gtpbp3^−/-^*zebrafish recapitulated the clinical phenotypes in HCM patients carrying the *GTPBP3* mutations. Our findings highlight the fundamental role of aberrant nucleotide modifications of mitochondrial tRNAs in mitochondrial biogenesis and their pathological consequences in hypertrophic cardiomyopathy.

## Supplementary Material

gkz218_Supplemental_Files
