# Supplementary material for: Deletion of Gtpbp3 in zebrafish revealed the hypertrophic cardiomyopathy manifested by aberrant mitochondrial tRNA metabolism
Source: Nucleic Acids Res. 2019 Mar 27;47(10):5341–55. doi: 10.1093/nar/gkz218 (PMC6547414; doi:10.1093/nar/gkz218)
Supplement: gkz218_Supplemental_Files [file gkz218_supplemental_files.pdf]

Supplementary data
Figure S1
Figure S2
Figure S3
Figure S4
Table S1
Table S2

Supplementary Figure S1

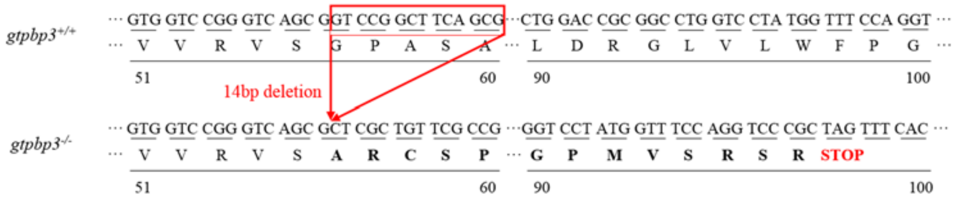


**Supplementary Figure S1. Partial sequences of *gtpbp3* cDNA and *gtpbp3*$^{K}$.**The c.167_180delGTCCGGCTTCAGCG (del14) mutation is indicated with a red box, and this frame shift mutation resulted in the p.Gly55Alafs*44 mutation in the amino acid sequence, leading to replacement of glycine with alanine at position 55, and a truncated protein with 98 amino acids.

Supplementary Figure S2

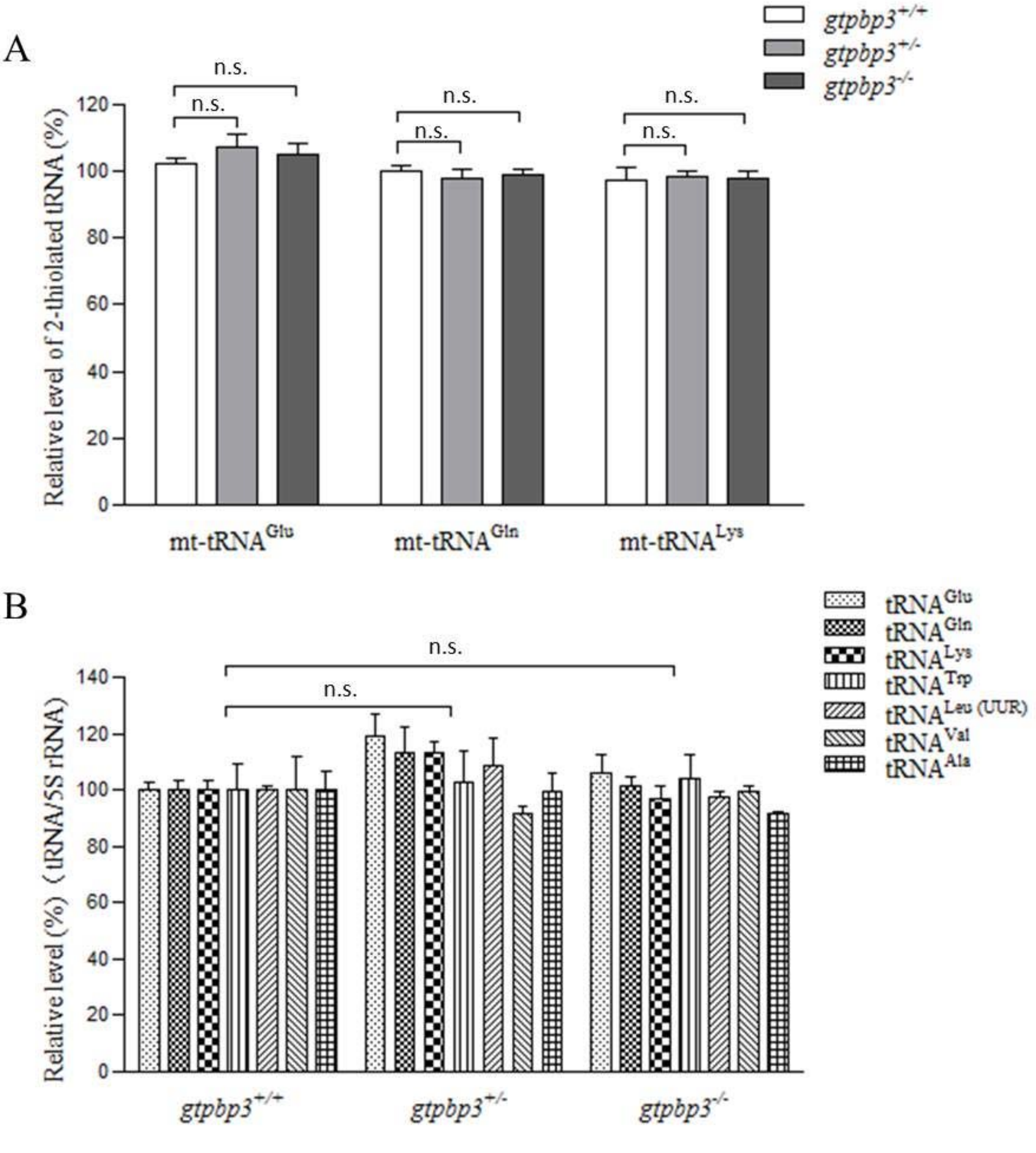


**Supplementary Figure S2. Quantification of the levels of mitochondrial tRNAs.** (A) Proportion in vivo of the 2-thiolated tRNA levels. The proportion values for the mutant zebrafish are expressed as percentages of the average values for the wild type zebrafish. The calculations were based on three independent determinations of each tRNA in each fish. The error bars indicate standard errors; p indicates the significance, according to Student's t test, of the difference between mutant and wild type for each tRNA. (B) Quantification of the levels of tRNAs. Average relative levels tRNA content were normalized to the average content in the mutant and wild type 5S rRNA, respectively. The values for the *gtpbp3*$^{+/-}$ and *gtbbp3*$^{-/-}$ zebrafish are expressed as percentages of the average values for the *gtpbp3*$^{+/+}$ zebrafish. The calculations were based on three independent determinations. Graph details and symbols are explained as above.

Supplementary Figure S3

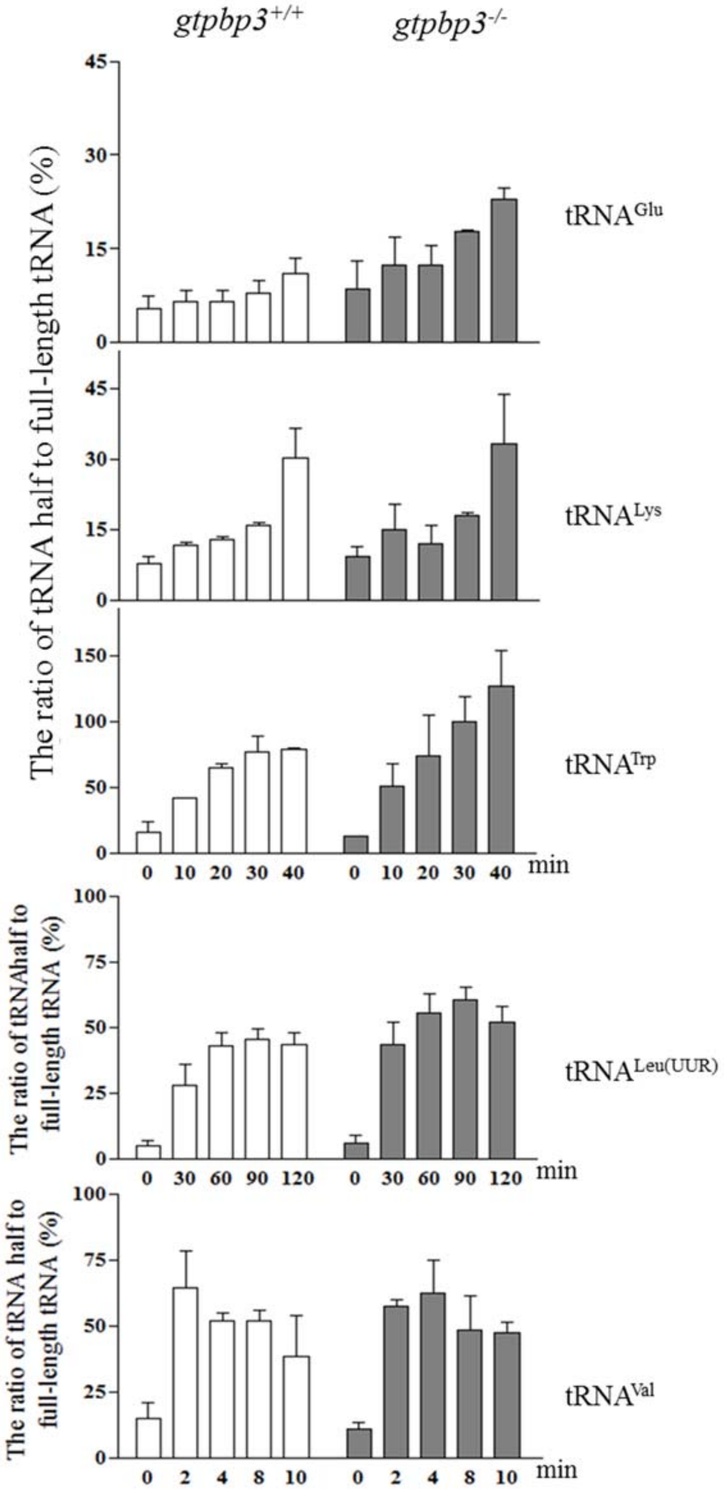


**Supplementary Figure S3. The ratios of tRNA half to full-length tRNA.** The ratios of tRNA half to full-length tRNA with S1 digestions of $tRNA^{Glu}$, $tRNA^{Lys}$, $tRNA^{Trp}$, $tRNA^{Leu(UUR)}$ and $tRNA^{Val}$, purified from *gtpbp3*$^{-/-}$ and WT zebrafish. The calculations were based on three independent determinations.

Supplementary Figure S4

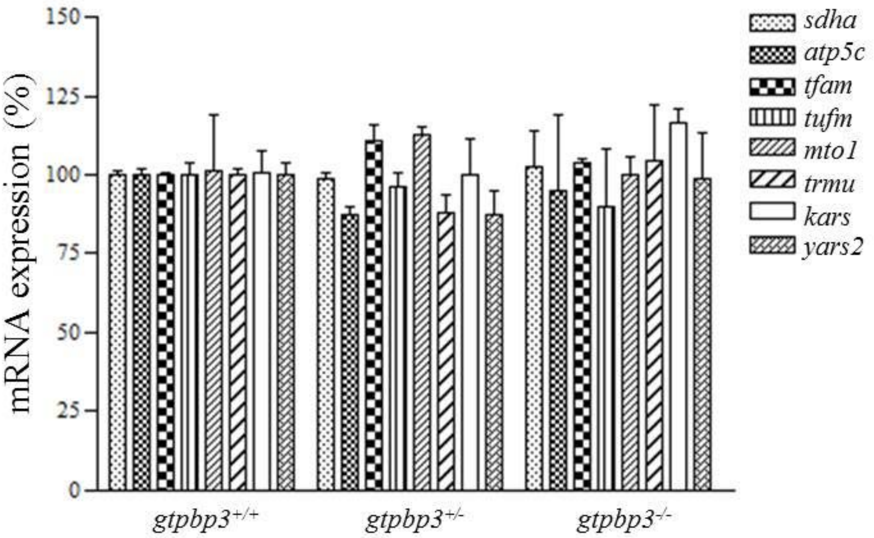


**Supplementary Figure S4. Relative gene expression levels of 8 nuclear genes encoding mitochondrial proteins.** Gene expression levels of *sdha*, *atp5c*, *tfam*, *tufm*, *mto1*, *trmu*, *kars* and *yars2* were normalized to the average levels of *gapdh* in mutant and wild type zebrafish, respectively.

**Supplementary table S1. Sequences of oligodeoxynucleosides for Northern blot analysis**

| tRNA | Sequences |
|---|---|
| mt-tRNA$^{Glu}$ | AATTCTTACTCAGACTTTAACTGAGACCGG |
| mt-tRNA$^{Gln}$ | TTAGAAAGAAGGGGGTCGAACCCATGCCCA |
| mt-tRNA$^{Lys}$ | TCACTAAGGGTGGTCGGTAAGCACCAAGTT |
| mt-tRNA$^{Trp}$ | TCTACTGAGAGCTTTGAAGGCTCTTGGTCT |
| mt-tRNA$^{Leu(UUR)}$ | GGCCTTTTGCAATTACCGAGCTCTGCCATC |
| mt-tRNA$^{Tyr}$ | TGGTAGAAAAAGGACTCAAACCTCTGTCCG |
| mt-tRNA$^{Met}$ | TGATGAAGGAGGGACTTTAACCATCATGTT |
| mt-tRNA$^{Ala}$ | TAGGACTTACAGACGTTACTCCGCATCTTC |
| mt-tRNA$^{Val}$ | CAGGACGATCCGATTTGCACGGATGTTTTC |
| 5S rRNA | GCAACCTAGTTTTCCCATGTGGTCTCCCAT |

Oligodeoxynucleosides used for DIG-labeled probes were zebrafish mitochondrial tRNAs (GeneBank accession No. NC_002333.2)

**Supplementary table S2. Sequences of oligodeoxynucleosides for real-time PCR analysis**

| Gene | Forward Primer | Reverse Primer |
|---|---|---|
| *sdha* | TGGTTATGGCCGCACATTCT | ATTCCTCCCTCTCCACGACA |
| *atp5c* | GTGTGGCCAAAGCCATCAAG | TGAAAGTAGGAGGCTTGCGG |
| *tfam* | CCCAAGGAGCACCTTCAACA | GTGCTCCTCCCACGATTTGA |
| *tufm* | CGCTGTGTGCTCTGGAAAAC | TAGACTCCGTCTACGGGCAT |
| *mto1* | CCGATAGGATTTCACTGTAACGC | ACCACCGCCAACCACAATTA |
| *trmu* | ACCGCATGCCTTTAACTCCA | TTGTGGGTCCCAGACGAATG |
| *kars* | GTGGTCGAACATTAGCCGGT | GCGCTTGCAGTTGGCATATT |
| *yars2* | GAGTCTCCACCGGATCTTCAC | GCCCATTCGAAAACTCCTGC |
| *gapdh* | TTCCAGTACGACTCCACCCA | TGACTCTCTTTGCACCACCC |
